# Phenanthrene Mitigates Cadmium Toxicity in Earthworms *Eisenia fetida* (Epigeic Specie) and *Aporrectodea caliginosa* (Endogeic Specie) in Soil

**DOI:** 10.3390/ijerph15112384

**Published:** 2018-10-27

**Authors:** Ali Mohamed Elyamine, Javaria Afzal, Muhammad Shoaib Rana, Muhammad Imran, Miaomiao Cai, Chengxiao Hu

**Affiliations:** 1 Key Laboratory of Arable Land Conservation (Middle and Lower Reaches of Yangtze River), Ministry of Agriculture, Research Center of Micro-elements, College of Resource and Environment, Huazhong Agricultural University, Wuhan 430070, China; elyoh@hotmail.fr (A.M.E.); juvaria_afzal@outlook.com (J.A.); muhammadshoaib@webmail.hzau.edu.cn (M.S.R.); imrangorayauaf@yahoo.com (M.I.); caimiaomiao@webmail.hzau.edu.cn (M.C.); 2 Hubei Provincial Engineering Laboratory for New Fertilizers, Huazhong Agricultural University, Wuhan 430070, China; 3 Faculty of Science and Technology, Department of Life Science, University of Comoros, Moroni 269, Comoros

**Keywords:** cadmium, phenanthrene, earthworms, toxicity, interaction effects, ecotype

## Abstract

In classical toxicology studies, the interaction of combined doses of chemicals with dissimilar modes of toxic action in soil is complex and depending on the end point investigated and the experimental protocol employed. This study was used to examine the interactive effect of phenanthrene and Cadmium on two ecologically different species of earthworms; *Eisenia. fetida* and *Aporrectodea. caliginosa*. This interactive effect was scrutinized by using the acute toxicity test with the concentrations of 2.51 mg kg^−1^ and 3.74 mg kg^−1^, respectively, being lethal for 50% of *E. fetida* and *A. caliginosa*. The results showed that in the mixture treatment, phenanthrene at 5, 10, 15 and 20 mg kg^−1^ significantly mitigated both earthworms species mortality and body-mass loss. Moreover, the factor of Cd accumulated in *E. fetida* and *A. caliginosa* tissues was significantly decreased by about 12% and 16%, respectively. Linear regression correlation coefficient revealed that the reduction of both earthworm species mortality was negatively and significantly correlated (*r*^2^ = 0.98 ± 0.40 and 1 ± 3.9 *p* < 0.001) with phenanthrene concentration in soil. However, over 20 mg kg^−1^ of phenanthrene, both organisms mortality rate increased again, as was the Bioaccumulation factor of phenanthrene. Thus, this study proposes that the antagonistical effect of phenanthrene on Cd at a degree of concentration can be used to mitigate Cd effect on soil living organisms. However, as an implication of these results, the interpretation of standardized toxicity bioassays, including whole effluent toxicity tests and single-compound toxicity tests, should be performed with caution. In addition, risk assessment protocols for environment pollution by a mixture of metals and polycyclic aromatic hydrocarbons should include robust methods that can detect possible interactive effects between contaminants to optimize environmental protection.

## 1. Introduction

The mixture of heavy metals and polycyclic aromatic hydrocarbons (PAHs) in classical toxicology studies or in remediation processes is gaining increasing interest in the scientific world [1,2]. Indeed, due to anthropogenic activities, namely the application of pesticides in agricultural soil [3], inorganic fertilizers [4], industries including metal-plating, mining, tannery, petrochemical, textile, battery and fertilizer production [5], discharge of waste containing heavy metal [6] and emission from industries production [7], agricultural soils contamination with heavy metals and PAHs have increasingly beoame of serious global environmental concern. This pollution poses a huge threat to human beings and natural ecosystems [8]. Cadmium (Cd), one of the most toxic pollutants because of its non-degradability, persistence in nature, and high toxicity to plants, soil organism and humans [9] is widely spread in the environment. It negatively affects plant physiological growth [10], soil organism biomass [11], and may affect human health through food-chain bioaccumulation [12]. Even though Cd is indestructible, Liu et al. [13] reported that it may become less toxic to the environment either by chelating with chelators via chemical or physical remediation, or by shifting the valence by redox reaction by forming interactions, in some specific cases, with PAHs. Phenanthrene, a three-fused ring compound commonly that is present in PAH-contaminated soils [14], is designed with less toxic effects compared to other degradable PAHs. Although it is not mutagenic or carcinogenic, phenanthrene has been reported to affect nitrifying bacteria in soil [15], reduce the intraradical colonization of arbuscular mycorrhizal fungi in maize root [16], inhibit the growth of algae [17] and, at a given concentration, can inhibit root exudation [18].

The joint toxicity of contaminants to organisms in their natural habitat can be complex. It may depend on the chemistries of the individual compounds, environment-specific bioavailability, toxicological mode of action, organism test used, experimental conditions and possible pharmacologic interaction among contaminants once bio-accumulated [19]. Therefore, the dose combination of two chemicals with a dissimilar toxic mode of action may cause synergistic, independent or antagonistic effects on tested organisms, depending on the end-point investigated and the experimental protocol utilized [20]. Lu et al. [21] found that the addition of a moderate dosage of pyrene could promote microbial prosperity in soil and, thus, relieve metal stress. While Gauthier et al. [22] summarized that the more-than-additive deleterious effects of PAHs–metal mixtures to soil organism were common in metal-PAHs mixtures. Gust and Fleeger [23] reported that the joint effect of phenanthrene and Cd on *Ilyodrilus templetoni* was antagonistic and phenanthrene reduced Cd toxicity on *Ilyodrilus templetoni*. In another study, they showed that phenanthrene increased the lethal toxicity of co-occurring Cd in *Hyalella Azteca* in sediment exposure [24]. Thus, it is obvious that the couple “phenanthrene-Cd could as well be more or less toxic to soil or aquatic organisms, compared to their effects in single exposure. Although the main mechanism of their interaction effect is still not deeply understood, studies showed that the interactive effect of phenanthrene and Cd was strongly concentration-dependent rather than being a toxic joint effect [25]. The combined dose of Cd and phenanthrene at, respectively, 3.2 mg kg^−1^ and 1.6 mg kg^−1^ induced high toxicity effect compared to the effect of their combination at 3.2 mg kg^−1^ and 25.6 mg kg^−1^, respectively [26]. Phenanthrene concentration at 25.6 mg kg^−1^ reduced significantly Cd toxicity by about 52% [27]. However, Zhu et al. [26] reported that the genotoxicity effect of the combined Cd and phenanthrene at, respectively, 50 mg kg^−1^ and 12.5 mg kg^−1^ was higher compared to the effect of either Cd or phenanthrene in single exposure. Therefore, there must be a range of concentrations by which phenanthrene could interact with Cd and mitigate its toxic effects on living organisms. Although different organisms reacted differently to an exposure stress situation, earthworms appeared to be excellent candidates for eco-toxicology studies. With their ability to accumulate essential and non-essential heavy metals in their body tissues and their direct or indirect role to modulate the transfer of organic and inorganic pollutants by virtue of their habitation [27], earthworms are considered as bio-indicators of contaminated soil and key diagnostic indices in eco-toxicology [28]. They ameliorate soil structure by increasing soil aeration [29,30] and enhance the conveyance of soil microorganisms.

Most previous studies on the toxicity of heavy metals and PAHs either in single or combined dose test to earthworms have been concentrated on appraising the concentration of the pollutants in earthworms and their effects on worm growth, biomass and reproduction. However, taking into account the effects of these pollutants, such studies indicated an approximate concentration of pollutants that adult earthworms can tolerate, but provide no indication of the actual toxicity of pollutants to earthworms and how the interaction of organic and inorganic pollutant should be controlled. Exploiting the antagonistic interaction of heavy metal and PAHs in eco-toxicology study would have a significant impact on the control of heavy metals toxicity and could provide clear evidence regarding to the threshold concentration of pollutants in a mixture treatment.

The purpose of this study is to examine the interactive effect of phenanthrene and Cd on two ecologically different species of earthworms; *E. fetida* (epigeic specie) and *A. caliginosa* (endogeic specie). The use of ecologically different earthworm species could be promising and effective, since they inhabit the soil at different depth, have different sizes and feeding habits. This paper further determines at a degree of concentration, the relational effect between cadmium and phenanthrene on earthworm life cycles including mortality and body weight variation.

## 2. Materials and Methods

### 2.1. Soil Properties

Soil was collected from the test field at Huazhong Agricultural University (HZAU) (30°28′26″ N, 114°20′51″ E). The upper litter was removed, and the soil from the top layer (0–20 cm) was collected. The soil samples were transferred to the greenhouse of Micro-element Research Center at HZAU for grinding and sieving. The used soil presented the following proprieties: pH (soil:H_2_O 1:2.5) 7.6; organic matter 1.31%; soil moisture content 18.58 ± 0.59%; NH_4_Cl exchangeable K, 127.99 mg kg^−1^; total nitrogen N 0.17%; Olsen-P of 39.69 mg kg^−1^; CEC 11.47 cmolc kg^−1^; and Ca, 2288.2 mg kg^−1^.

### 2.2. Test Soil Preparation

Three kg of air-dried soil was placed into ceramic pots. For cadmium concentration, a desired amount of CdCl_2_ (98%, purity) was dissolved in aqueous solution. This solution was poured on the soil surface and the soil matrix was mixed thoroughly and incubated at 20 ± 1 °C for three months. Throughout the incubation time, the content of moisture was scrutinized each week and maintained by watering with DI water if needed.

Phenanthrene (97% purity) was dissolved in acetone (analytically pure) and the solution was thoroughly mixed with the soil to produce a final concentration of 5, 10, 15, 20, 25 and 30 mg kg^−1^ of phenanthrene respectively denoted as P5, P10, P15, P20, P25 and P30. The control treatment was prepared using clean soil spiked acetone only. The fresh spiked soils were stored in open containers in a fume hood until all of the solvent (acetone) evaporated.

### 2.3. Test Organism

Earthworm species *Aporrectodea caliginosa* and *Eisenia fetida,* two different ecotypes (endogeic and epigeic respectively) were selected for this study. A litter-dwelling *E. fetida* was chosen for its fast growth and its rapid productivity [31], while the horizontally burrowing mineral soil feeder *A. caliginosa* was selected for its ability to transfer nutrients or chemicals elements within a compartment of an ecosystem or between different compartments. [32]. Both earthworm species were chosen and selected in the earthworms breeding site at HZAU. A sufficient quantity of earthworms (for different species) was initially purchased from a commercial source and transferred to the greenhouse where a controlled earthworm breeding site containing soil filled with household waste (cabbage waste, carrot peelings, banana waste) was installed. The worms used in the present study were selected after 3 months of reproduction in this mentioned site, washed in deionized water, placed on wet filter paper and maintained in the darkness at 20 ± 2 °C for one night in the laboratory [33] before placing them in the surface of the corresponding experimental pot.

### 2.4. Acute Toxicity Test

Preliminary studies were carried out to assess the range of Cd concentrations that produced 1–100% mortality. Thus, five concentrations in a geometric series and a control treatment were used to get the concentration value that exhibited 50% mortality (LC50) at 95% confidence interval. Cd concentrations used in the single chemical toxicity experiment were as: 0.5, 1, 3, 5 and 10 mg Cd per kg of soil (dry weight). The choice of the interval of Cd concentration was made to ensure a relatively high effect in the highest concentration and no observable effect in the lowest concentration.

Contrary to Cd, preliminary experiment was realized to evaluate the range of phenanthrene concentration that could at a time produce low observable effect on the tested organism in the highest concentration and have a mitigating effect on Cd toxicity. The concentrations of phenanthrene used were 5, 10, 15, 20, 25 and 30 mg kg^−1^.

### 2.5. Toxicity Test with Chemicals Mixture

The effect of Cd in the mixture treatments were based on the LC50 values obtained earlier. Six concentrations of phenanthrene with separation factor of 5 were used to set up the mixture treatment with LC50 of each species. Each treatment was tested in three independent experiments with replicates samples. For each specie, six binary mixtures (CdP5, CdP10, CdP15, CdP20, CdP25 and CdP30) were tested to assess the impact of phenanthrene on Cadmium effect.

### 2.6. Experimental Monitoring

For each ecotype, 20 adults and healthy individuals with similar fresh weight (1.82 ± 0.06 g and 3.09 ± 0.04 g respectively for *E. fetida* and *A. caliginosa*) were randomly selected in the earthworms breeding site at HZAU, regrouped and rinsed with DI water and placed on moist paper for 24 h to void the gut content before placing them on the soil surface of each experimental pot previously mixed with horse dung (3:2) as food. The initial body weight was measured immediately (day-0) and the animals were placed on the pot experiment. The experiment was carried out for 30 days period during which the mortality and the body mass of earthworms was monitored every 5 days. Earthworm was considered dead when it did not show any response on probing.

### 2.7. Kinetics Parameters

Bioaccumulation factor (BAF), uptake constant rate (*k*_1_) and elimination constant rate (*k*_2_) were monitored to understanding whether there was correlation between earthworm mortality and body mass variation. Every fifth day three individual earthworms for each specie were sorted from each test soil and starved on moist paper for 24 h (elimination phase) to allow them void their gut content. The paper was changed twice during the starvation period (at 6 and 18 h). After starvation, the worms were kept at −28 °C for further analysis [34]. The gut content was then collect and use to analysis chemical concentration. BAF was calculated as the ratio of the pollutant content in the earthworm tissue to that in the corresponding soil [35]. Elimination constant rate (*k*_2_) was determine as the concentration eliminated per day and the uptake constant rate (*k*_1_) was calculated by using the formula described by [36,37]: *k*_1_ = BAF × *k*_2_

### 2.8. Chemical Analysis

Earthworms were washed in deionized water and placed on wet filter paper to allow depuration for 48 h. Earthworms were then washed again, freeze-dried and ground. A sub-sample of 500 mg was used for chemical analysis.

#### 2.8.1. Cadmium

Earthworm sample was digested with 6 mL of HNO_3_/H_2_O_2_ mixture (5:1) on a hot plate at 150 °C for 2 h [37]. The digested solution was evaporated to 1 mL and 1% HNO_3_ was added to adjust the volume to 25 mL. The diluted solution was filtered, and Cd concentrations were measured by atomic absorption spectrometry (AAS) (Z-2000, HITACHI, Tokyo, Japan).

#### 2.8.2. Phenanthrene Determination

Ultra-sonication extraction method [38] with slight modification combined the protocol described in [39,40] was used to determine phenanthrene concentration. Frozen worms were ground and mixed with 1.5 times its wet weight of Na_2_SO_4_ to a fine powder. The mixture was extracted with 10 mL of acetone placed in ultrasonic bath with ice water for 30 min. The solution was shaken for 1 min, resonicated for 30 min and centrifuged at 13,700× *g* for 15 min; the whole process was repeated twice. Further chemical treatments which the detail of the method is described in [39] were performed before determining phenanthrene concentration on High-performance liquid chromatography (HITACHI Chromaster 5300, Hitachi Beijing Tech Information Systems Co., Ltd, Beijing, China.

The accuracy and analysis quality of phenanthrene measurements was checked by using certified standard materials NIST1647 Priority Pollutant Polycyclic Aromatic Hydrocarbons in Acetonitril, purchased from Sigma-Aldrich and the recovery rate was 85 ± 3%.

### 2.9. Statistical Analysis

All data were subjected to the Analysis of Variance (ANOVA) using Statistical Package for Social Science (SPSS. 20, IBM Company, Chicago, IL, USA) statistical software following by Post hoc Dunnett multiple comparison test and Tukey comparisons test with 95% confidence level to compare the means. Linear regression was used to find out the correlation between concentration of pollutant and different parameters. Cd LC50 mortality and the dose-mortality response were analyzed by probit analysis [41]. The 30-d LC50 was 2.51 mg kg^−1^ and 3.47 mg kg^−1^, respectively, for *E. fetida* and *A. caliginosa*. Different graphs were performed using Origin (8 Pro SR4, OriginLab (Guangzhou) Ltd., Guangzhou, China) software.

## 3. Results

### 3.1. Response of Earthworms to Individually Chemicals

#### 3.1.1. Mortality

**Figure 1:**
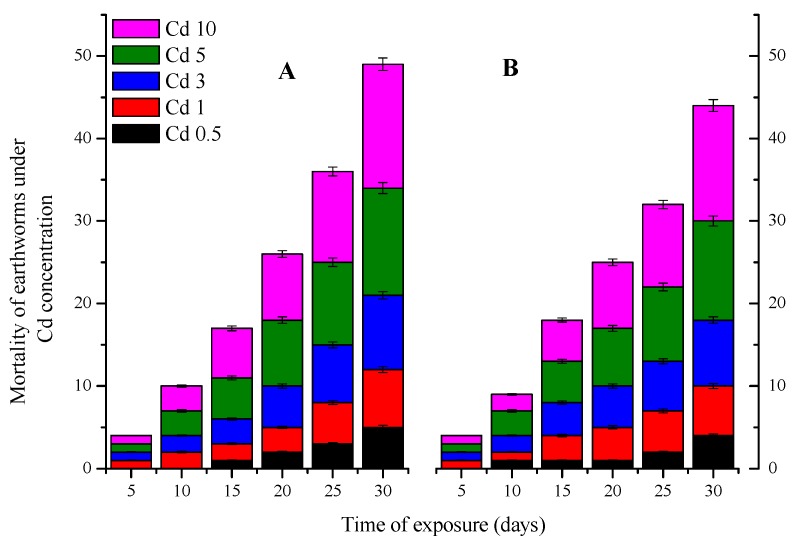
Mortality of *E fetida* (**A**) and *A. caliginosa* (**B**) under Cd stress at different concentration (0.5, 1, 3, 5 and 10 mg kg^−1^).

Throughout the experimental period, no earthworms died in both experimental controls. However, compared to the control, significant differences were registered regarding to the mortality rate of both *E. fetida* (Figure 1A) and *A. caliginosa* (Figure 1B) among the different Cd-contaminated soils. Thereby, linear regression correlation coefficient (*r*-value) indicated that the mortality of both worms species was significantly correlated with “Cd inoculation” factor (*p* = 0.01, *r*^2^ = 0.9 ± 0.19, and 0.89 ± 0.27 respectively for *E. fetida* and *A. caliginosa*) and “time of exposure” factor (*p* = 0.02, *r*^2^ = 0.98 ± 0.4 and 0.99 ± 1.8).

**Figure 2:**
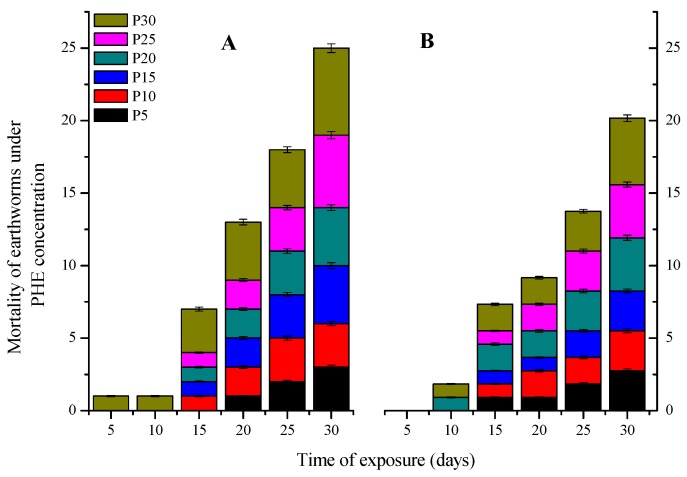
Mortality of *E fetida* (**A**) and *A. caliginosa* (**B**) under phenanthrene stress at different concentration (5, 10, 15, 20, 25 and 10 mg kg^−1^).

The mortality effect of phenanthrene on earthworms, compared to that of Cd, in all treatments was low with a maximum percentage of 30% at P30 treatments for *E. fetida* (Figure 2A) and 25% for *A. caliginosa* (Figure 2B). Despite this low mortality effect caused by phenanthrene, the linear regression correlation coefficient value on both earthworms mortality was >0.92 ± 2.16 indicating positive and significant interaction between phenanthrene concentrationa in soil and earthworm mortality.

#### 3.1.2. Body Weight Variation

Cd, as well as its concentration increased in soil; the body weight of both earthworms species decreased significantly compared to the control. In Table 1 is summarized the effect of LC50 values on both earthworms species body mass variation with respect to the time. The cumulative mean of the body weight loss (one-way ANOVA, *p* < 0.05) was 0.47 ± 0.5 corresponding to 11% for *E. fetida* and 0.52 ± 0.6 corresponding to 7% for *A. caliginosa* after 30 exposure days. The correlation test showed that the loss of earthworm weight (*r*^2^ = 0.89 ± 0.09 and 0.91 ± 4.04, *p* < 0.001) correlated with the mortality rate of earthworms.

### 3.2. Responses of Earthworms to Chemical Mixture

#### 3.2.1. Mortality

**Figure 3:**
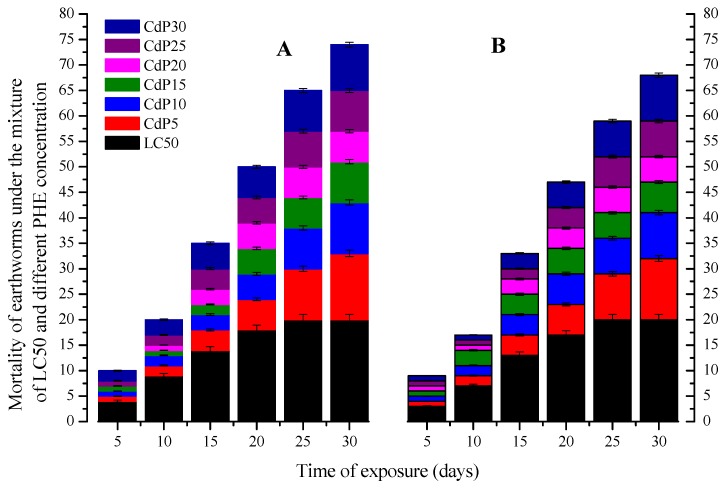
Mortality effect of LC50 values in the mixture treatment with phenanthrene different concentration (5, 10, 15, 20, 25 and 10 mg kg^−1^) on *E fetida* (**A**) and *A. caliginosa* (**B**).

In the combined dose treatments, from CdP5 to CdP20 treatments, both earthworms species mortality was significantly reduced compared to LC50 effect at single exposure (Figure 3). The correlation test showed that this reduction had negative and significant correlation (*r*^2^ = −0.98 ± 0.4 and 1 ± 3.97 *p* < 0.001) with the extracted phenanthrene concentration in soil. However, from CdP25 to CdP30, both earthworm species mortality increased slightly compared to that registered in CdP10 to CdP20 treatments. This indicated that 20 mg kg^−1^ is probably the threshold phenanthrene concentration to counteract the negative effects of Cd LC50 values.

#### 3.2.2. Body Mass Variation

The concentration responses generated by the interaction of the LC50 values and different concentration of phenanthrene on the body weight variation rate of both earthworm species are represented in Table 2. The combined dose enabled us to gain a cumulative mean of body weight of 3% and 2%, respectively, for *E. fetida* and *A. caliginosa*, compared to their body weight loss induced by LC50 alone. The linear regression correlation coefficient indicated that, from CdP5 to CdP15 treatments, the cumulative loss of *E. fetida* body weight was negatively and significantly correlated (*r*^2^ = 0.99 ± 0.37, *p* = 0.022) with the extracted phenanthrene concentration in soil, while from CdP20 to CdP30 it was positively correlated (*r*^2^ = 0.99 ± 1.54, *p* = 0.012) with the corresponding phenanthrene concentration in soil. Contrariwise, the cumulative loss of *A. caliginosa* body mass from CdP5 to CdP25 was highly significant and negatively (*r*^2^ = 0.83 ± 1.51, *p* < 0.001) correlated with the phenanthrene concentration in soil. However, at CdP30 treatment the loss of body weight increased slightly while remaining lower than that induced by LC50 alone.

### 3.3. Chemicals Accumulation in Earthworms

#### 3.3.1. Single Exposure Treatments

##### Cadmium

The mean concentrations of Cd expressed as mg kg^−1^ dry weight for the both earthworms were investigated. Cd concentration in both earthworm species was significantly different among the different treatments. Despite the fact that Cd concentration in both earthworms species tissues clearly increased with the exposure to raised soil Cd concentration, the accumulation in *A. caliginosa* was higher than that in *E. fetida*. Thereby, to depict whether earthworms mortality and the change in their body mass was related to Cd concentration or/and time of exposure, BAF, the uptake rate (*k*_1_) and elimination rate (*k*_2_) constant in earthworm body tissues were estimated and presented in Table 3. BAF in both earthworms decreased gradually with the increase of Cd concentration in soil. No significant differences were observed in the Cd uptake rate constant (*k*_1_) in both earthworms] species within an exposure level (ANOVA, Tukey’s test). However, with respect to each earthworm’s specie, the corresponding parameters values increased with elevated exposure level. Contrary to the uptake constant, the elimination rate constant (*k*_2_) of both earthworms was significantly different.

BAF of Cd in both earthworms species estimated in an experiment with LC50 values as a function of time was presented in Table 4. Beside the fact that Cd accumulation in earthworms was highly significant (*p* <0.001) and correlated with the time of exposure (*r*^2^ = 0.80 ± 2.5 and 0.96 ± 0.57), both earthworms species mortality was significantly correlated to Cd accumulated in worms (*r*^2^= 0.81 ± 2.16, *p* = 0.02 and *r*^2^ = 0.72 ± 2.41, *p* = 0.03 respectively for *E. fetida* and *A. caliginosa*).

##### Phenanthrene

Phenanthrene concentration accumulated in both earthworm species tissues was not significantly different (Table 5). Although, the uptake rate constant (*k*_1_) and the elimination rate (*k*_2_) constant in both earthworms species increased with elevated exposure level of phenanthrene in soil, no significant differences were observed among the two species (ANOVA, Tukey’s test, *p* = 0.05). With respect to the concentration of phenanthrene in soil, BAF of both earthworm species gradually decrease within the increase of the corresponding concentration in soil.

#### 3.3.2. Combined Dose Treatments

**Figure 4:**
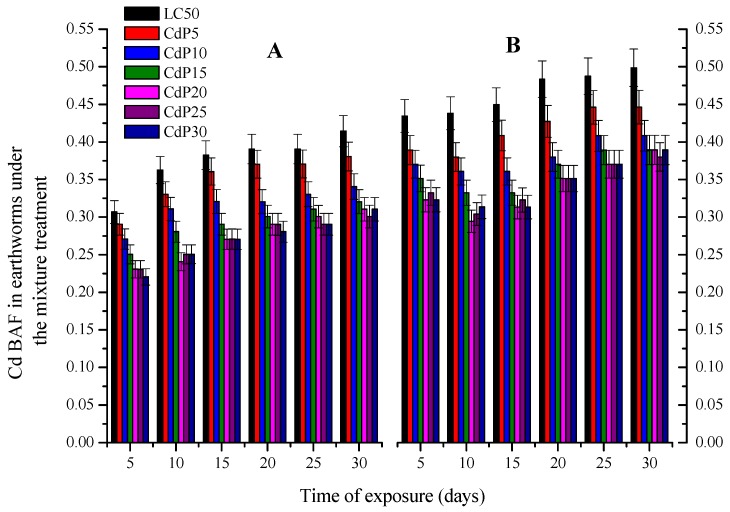
Bioaccumulation factor (BAF) of Cd on *E fetida* (**A**) and *A. caliginosa* (**B**) under the mixture treatment with different phenanthrene concentration with the respect to the time. Different represented bands are means of three replicates with standard error (Tukey’s Studentized Range HSD test *p* = 0.05).

Means of Cd concentrations accumulated in both earthworm species measured in Cd-phenanthrene mixture treatments were reported in Figure 4. The BAF was found to be a function depending not only on the exposure time (*r*^2^ = 0.93 ± 0.53, and 0.80 ± 0.34, *p* <0.001) but also was negatively correlated with the concentration of phenanthrene in the soil (*r*^2^ = 0.84 ± 0.37 and 0.79 ± 0.40, *p* <0.001 respectively for *E. fetida* and in *A. caliginosa*). The cumulative mean of Cd accumulated in *E. fetida* and in *A. caliginosa* tissues was significantly (*p* <0.001) reduced by about 0.25 ± 0.05 mg kg^−1^ corresponding to 12% and 0.33 ± 0.04 mg kg^−1^ corresponding to 16%, respectively (Figure 4).

**Figure 5:**
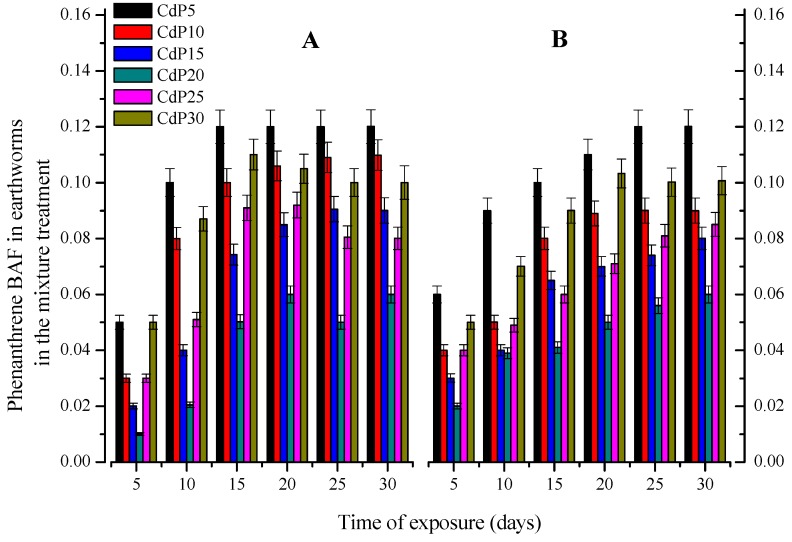
Phenanthrene Bioaccumulation factor (BAF) on *E fetida* (**A**) and *A. caliginosa* (**B**) under the mixture treatment of different phenanthrene concentration and Cd LC50 values with the respect to the time. Different presented bands are means of three replicates with standard error (Tukey’s Studentized Range HSD test *p* = 0.05).

The mean of phenanthrene concentration accumulated in both earthworm species is represented in the Figure 5. For both species, phenanthrene BAF was negatively proportional to its concentration in soil at CdP5 to CdP20. However, over 20 mg kg^−1^, the concentration accumulated by both earthworm species recovered and in most of case was even higher compared to that in CdP10 and CdP15. This suggested that the increase of both earthworm species mortality rate in CdP25 and CdP30 treatments described above could be related to phenanthrene effects or to their interaction with Cd rather than Cd effects.

## 4. Discussion

### 4.1. Mortality in Earthworms

Throughout the experimental period, the high survival rate observed for both earthworm species in the control treatments suggests that the experimental conditions were acceptable in terms of providing a vital environment and suitable media for earthworm survival. The toxicity of a chemical to earthworms depend on the feeding strategies adopted by the species of earthworms and moreover on the availability of the pollutant [42]. Cd is well-known as the metal with the most adverse effects on soil living organisms, including earthworms [9]. The pattern of mortality suggests that the effects of pollutants on earthworms were as a result of absorption and/or uptake of the chemicals across the worm body, through either body wall or by ingestion. In this present study, Cd at 2.51 and 3.47 mg kg^−1^ exhibit 50% of mortality response to *E. fetida* and *A. caliginosa* within 11 and 13 days, respectively. Cd seemed to be highly toxic to earthworms; certain others heavy metals such as Cu, Ni and Zn have been reported to be more toxic [27]. The difference between these two types of metals could be explained by the fact that Cd as non-essential metal necessitates a period of time to let the toxic symptoms develop, while with the essential metals such as Cu in most organisms, the range between deficiency and toxicity is low especially in the species like earthworms which are not equipped with effective barriers to control the absorption of the pollutant.

Phenanthrene, a certain concentration affected both earthworms; compared to Cd exposure effects, its effect in all treatments was low with a maximum percentage of 30% for *E. fetida* and 25% for *A. caliginosa*. This result may be explained by the low range of its contamination. Anyanwu and Semple [43] reported that 21-d LC50 and EC50 (based on weight loss) of phenanthrene to *E. fetida* ranged from 400 to 500 mg kg^−1^ dry soil and 1.2–500 mg kg^−1^ dry soil, respectively. Wu et al. [44] calculated the 14-d LC50 of phenanthrene as 40.67 mg kg^−1^ and reported that the concentration and the duration of exposure had a significant effect on earthworm growth, mortality and body weight.

In the combined dose treatments, phenanthrene reduced the lethality of both earthworms (Figure 3). This suggested that phenanthrene affected Cd and alleviated its toxicity on earthworms. Gust and Fleeger, [23] reported that in the of exposure Cd-phenanthrene mixture, phenanthrene induce modification in Cd assimilation efficiency, its uptake and efflux rate. Although there is no evidence to provide a general conclusion regarding how organic contaminants affect metal activities, Moreau et al. [45] reported that phenanthrene alleviated Cd toxicity by diminishing Cd bioaccumulation rate, or by modifying its bioavailability. Gorria et al. [46] also reported that phenanthrene may inhibit the absorption of Cd by altering the membranes fluidity and changing its electrical potential. However, the reduction of both earthworms’ mortality from CdP5 to CdP20 and its slight increase from CdP25 to CdP30, we propose that whatever the modality and mechanism by which phenanthrene interacts with Cd to counteract its effects, there must be a sort of interaction threshold by which high above, the mixture phenanthrene-Cd could cause non-intentional effects on the organism test. If in the present study, it was found that over 20 mg of phenanthrene kg^−1^ of soil, the toxicity effect of the couple phenanthrene-Cd increased, it is to highlight that the consequences could not be limited only to earthworms, but could especially severely affect soil microorganisms. Indeed, it is well-known that the remediation of phenanthrene involved soil microbial. However, heavy metals can adversely compromise microbial activity by affecting ATP production, C-mineralization and, therefore, challenge the removal of phenanthrene [47]. In return, whilst phenanthrene is metabolized by Cytochrome P450, their intermediates such as salicylic acid can impact the microbial cell metabolism and thus affecting the absorption capacity of heavy metals [48].

### 4.2. Bioaccumulation and Earthworms Mortality

Soil-dwelling earthworms are recognized to uptake metals by two different pathways: (1) direct dermal contact with metal in soil and (2) intestinal pathway by ingestion of bulk soil [49] and accumulate it into their body tissue. Cd accumulation and earthworms mortality were significantly correlated (*r*^2^ = 0.81 ± 2.16, *p* = 0.02 and *r*^2^ = 0.72 ± 2.41, *p* = 0.03 for both species). This pointed out that, although the higher faculty of earthworms to absorb and accumulated heavy metal in their body tissues, at a given concentration when the metal uptake by an individual worm reaches a distinct threshold level, it may be toxic to worms. Wu et al. [50] reported that the metals distribution and their availability determine their toxicity to any living organism in soil. Thereby, earthworms developed a strategy by which, when the uptake chemical reaches a certain concentration level, the worms actively could eliminate the excess of pollutant in their corresponding cast [35]. This implies an uptake/elimination process based on the transport of metals into internal storage compartments and its excretion thereafter [51]. BAF for both worm species gradually decreased with the increase of Cd concentration in soil (Table 4). This result supports the observation by which, at low Cd concentration, earthworms absorb and accumulate Cd in their tissues. On the other hand, at higher concentrations, earthworms could absorb Cd but not accumulate it, and thus, eliminated Cd into their cast [52]. This could explain why the elimination rate constant *k*_2_ in both earthworm species was different from the uptake rate constant *k*_1_ (Table 4). However, the difference observed among the two species to uptake and eliminate Cd, could be related to their ecologically category. Maleri et al. [51] reported that *A. caliginosa* and *E. fetida* present different mechanism to accumulate and eliminate metal in their body tissues. In *E. fetida,* metal was equally distributed over the whole body, while in *A. caliginosa,* it was accumulated from the anterior to the posterior alimentary canal (PAC).

The low range of phenanthrene concentration accumulated in both worm species (Figure 5) indicates a degradation, sorption and/or transformation. Indeed, earthworms through cast production activities, upsurge the microbial population and circulation, which are the key drivers for the degradation and removal of organic pollutants in soil [29,53]. The presence of phenanthrene resulted in a significant reduction of Cd accumulation in both earthworm species (Figure 4). This confirms the result found by [43], whih reported that phenanthrene diminished Cd bioaccumulation rate by modifying its bioavailability and altering other parameters in the biodynamic model that contributes to the Cd bioaccumulation and Cd bioavailability. Chemically, phenanthrene with their benzene structure can strongly form complexes with heavy metals by non-covalent bridges, due to electrostatic attractions between the benzene quadripete and the positive charges of the metal cations, either by bio-molecular recognition or protein-ligand or others processes [13]. Relying on this hypothesis, we can speculate that the interaction of both chemicals could relate to their chemical structure and function. However, over 20 mg kg^−1^ of phenanthrene, the quantity of phenanthrene accumulated in worms increased (Figure 5), while that of Cd indicated no significant differences among all treatments (Figure 4). This suggested that in the mixture treatments, the mortality of both earthworms at CdP25 and CdP30 was probably caused by phenanthrene, rather than Cd.

### 4.3. Body Weight Variation

Body weight variation was a very sensitive parameter in determining the effect of Cd and phenanthrene at the tested concentrations on earthworms. In the single treatment with Cd, after 30 experimental days, *E. fetida* and *A. caliginosa* lost, respectively, 0.47 ± 0.5 corresponding to 11% and 0.52 ± 0.6 corresponding to 7% of their body weight. Generally, the loss of body weight induced by pollutants in different Cd and phenanthrene treatments is probably related to the earthworm’s strategy once exposed into stress condition, which is reducing food intake to avoid the toxins [54]. This strategy is commonly used by earthworms to avoid poisoning, not only by heavy metals but also by organic chemicals [55]. Indeed, once exposed to a polluted environment, earthworms are able to attenuate the toxin and its resulting effects by regulating their internal biochemical activities including the loss of their body weight. Cd-phenanthrene interaction on both earthworm species biomass was remarkably beneficial. The combined dose enabled to gain a cumulative mean of body weight of 3% corresponding to 0.27 ± 0.03 and 2% corresponding to 0.35 ± 0.05, respectively, for *E. fetida* and *A. caliginosa* species, compared to the body weight loss induced by LC50 alone (Table 2). These results were consistent with those found by Liu et al. [13], suggesting that phenanthrene affected Cd partitioning at the site of toxic action and its bioaccumulation.

## 5. Conclusions

This present study was carried out to investigate the effect of phenanthrene on Cd and their interaction on earthworm mortality and body weight variation in soil. The chemical structure of both pollutants may promote them to interact and form a complex which mitigate Cd toxicity and reduce its bioaccumulation in worms. Our study shows that at a degree of concentration, phenanthrene could antagonistically affect Cd on different ecological species of earthworms in soil and could render Cd less-toxic to earthworms. However, since there is evidence that the couple “phenanthrene-Cd” could also be more toxic to soil organisms, compared to their respective effects in single exposure, effluent toxicity tests and single compound toxicity tests should be performed with caution. As it is quite possible that the pollutant behavior and bioavailability changes depend on physicochemical edaphic interaction, soil type and others soil properties, further research involving these cited variables is needed to extend the result of the present study.

## Figures and Tables

**Table 1:** Effect of Cd LC50 values on earthworm body weight loss with the respect to the time.

Time	d-0	d-5	d-10	d-15	d-20	d-25	d-30
*E. fetida*	1.84 ± 0.5 ^a^	1.83 ± 0.6 ^a^	1.72 ± 0.5 ^b^	1.71 ± 0.6 ^b^	1.67 ± 0.4 ^c^	1.65 ± 0.4 ^c^	1.64 ± 0.7 ^c^
*A. caliginosa*	3.03 ± 0.5 ^a^	3.01 ± 0.5 ^a^	2.98 ± 0.4 ^b^	2.94 ± 0.5 ^bc^	2.94 ± 0.6 ^bc^	2.91 ± 0.6 ^bc^	2.82 ± 0.5 ^c^

Values presented are means of three replicates with standard error (Tukey’s Studentized Range HSD test *p* = 0.05). Different letters a, b, c …in the same row (7 values) indicate significant difference.

**Table 2:** Effect of Cd LC50 values under different phenanthrene concentration on earthworm body weight loss with the respect to the time.

***Eisenia fetida* Weight (g)**							
**Time**	**d-0**	**d-5**	**d-10**	**d-15**	**d-20**	**d-25**	**d-30**
**CdP5**	1.84 ± 0.5 ^a^	1.82 ± 0.6 ^a^	1.74 ± 0.4 ^b^	1.71 ± 0.6 ^b^	1.67 ± 0.5 ^c^	1.66 ± 0.4 ^c^	1.65 ± 0.8 ^c^
**CdP10**	1.83 ± 0.4 ^a^	1.81 ± 0.5 ^a^	1.72 ± 0.6 ^b^	1.73 ± 0.7 ^b^	1.68 ± 0.6 ^c^	1.66 ± 0.5 ^c^	1.65 ± 0.6 ^c^
**CdP15**	1.84 ± 0.5 ^a^	1.82 ± 0.7 ^a^	1.74 ± 0.4 ^b^	1.72 ± 0.5 ^b^	1.67 ± 0.5 ^c^	1.67 ± 0.6 ^c^	1.65 ± 0.5 ^c^
**CdP20**	1.83 ± 0.6 ^a^	1.83 ± 0.6 ^a^	1.73 ± 0.6 ^b^	1.72 ± 0.8 ^b^	1.68 ± 0.6 ^c^	1.66 ± 0.8 ^c^	1.65 ± 0.4 ^c^
**CdP25**	1.84 ± 0.8 ^a^	1.82 ± 0.5 ^a^	1.74 ± 0.5 ^b^	1.71 ± 0.5 ^b^	1.67 ± 0.5 ^c^	1.65 ± 0.5 ^c^	1.65 ± 0.6 ^c^
**CdP30**	1.84 ± 0.6 ^a^	1.82 ± 0.5 ^a^	1.72 ± 0.6 ^b^	1.70 ± 0.6 ^b^	1.67 ± 0.5 ^c^	1.65 ± 0.5 ^c^	1.64 ± 0.5 ^c^
***Aporrectodea caliginosa* Weight (g)**							
**CdP5**	3.03 ± 0.5 ^a^	3.01 ± 0.6 ^a^	2.98 ± 0.5 ^a^	2.94 ± 0.8 ^b^	2.94 ± 0.5 ^b^	2.91 ± 0.6 ^bc^	2.83 ± 0.5 ^c^
**CdP10**	3.02 ± 0.4 ^a^	3.01 ± 0.5 ^a^	2.98 ± 0.6 ^a^	2.95 ± 0.5 ^b^	2.95 ± 0.5 ^b^	2.92 ± 0.5 ^bc^	2.83 ± 0.6 ^c^
**CdP15**	3.02 ± 0.6 ^a^	3.02 ± 0.6 ^a^	2.99 ± 0.5 ^a^	2.95 ± 0.6 ^b^	2.95 ± 0.6 ^b^	2.92 ± 0.5 ^bc^	2.83 ± 0.6 ^c^
**CdP20**	3.03 ± 0.6 ^a^	3.02 ± 0.5 ^a^	2.99 ± 0.5 ^a^	2.95 ± 0.4 ^b^	2.95 ± 0.6 ^b^	2.92 ± 0.5 ^bc^	2.84 ± 0.7 ^c^
**CdP25**	3.02 ± 0.7 ^a^	3.02 ± 0.7 ^a^	2.99 ± 0.6 ^a^	2.96 ± 0.6 ^b^	2.96 ± 0.5 ^b^	2.93 ± 0.4 ^bc^	2.84 ± 0.5 ^c^
**CdP30**	3.03 ± 0.3 ^a^	3.02 ± 0.5 ^a^	2.99 ± 0.7 ^a^	2.95 ± 0.4 ^b^	2.97 ± 0.8 ^b^	2.92 ± 0.5 ^bc^	2.84 ± 0.6 ^c^

Values presented are means of three replicates with standard error (Tukey’s Studentized Range HSD test *p* = 0.05). Different letters a, b, c …in the same row (7 values) and in the same column (6 values) indicate significant difference.

**Table 3:** Cd Bioaccumulation factor (BAF), uptake rate (*k*_1_) and elimination rate (*k*_2_) constants in the both earthworms exposed to different Cadmium concentrations.

Treatment	*Eisenia fetida*			*Aporrectodea caliginosa*		
	BAF	*k*_1_ (d^−1^)	*k*_2_ (d^−1^)	BAF	*k*_1_ (d^−1^)	*k*_2_ (d^−1^)
**Cd0.5**	1.31 ± 0.3 ^a^*	0.024 ± 0.018 ^a†^	0.018 ± 0.06 ^d‡^	1.72 ± 0.4 ^a^*	0.022 ± 0.02 ^a†^	0.013 ± 0.04 ^d†^
**Cd1**	1.20 ± 0.5 ^a^*	0.05 ± 0.020 ^a†^	0.041 ± 0.04 ^c†^	1.58 ± 0.2 ^a^*	0.030 ± 0.02 ^a†^	0.019 ± 0.01 ^c‡^
**Cd3**	0.39 ± 0.3 ^b^*	0.02 ± 0.021 ^a†^	0.048 ± 0.07 ^c†^	0.49 ± 0.1 ^b^*	0.010 ± 0.10 ^ab†^	0.021 ± 1.02 ^bc†^
**Cd5**	0.28 ± 0.2 ^c^*	0.021 ± 0.02 ^a‡^	0.073 ± 0.01 ^b†^	0.31 ± 0.1 ^c^*	0.012 ± 0.10 ^ab‡^	0.041 ± 1.01 ^b†^
**Cd10**	0.22 ± 0.3 ^d^*	0.018 ± 0.06 ^ab‡^	0.084 ± 0.02 ^a†^	0.27 ± 0.2 ^d^*	0.017 ± 0.24 ^a‡^	0.063 ± 1.22 ^a†^

Values presented are means of three replicates with standard error (Tukey’s Studentized Range HSD test *p* = 0.05). Different letters a, b, c …in the same column (5 values), number of asterisk * in the same row (2 values (BAF only)) and different symbols ^†^ and ^‡^ in the same row (4 values) indicate significant difference.

**Table 4:** Cd LC50 bioaccumulation factor (BAF) on earthworms with the respect to the time.

Time	d-5	d-10	d-15	d-20	d-25	d-30
*E. fetida*	0.30 ± 0.67 ^b^	0.36 ± 0.55 ^ab^	0.38 ± 0.47 ^a^	0.39 ± 0.48 ^a^	0.39 ± 0.43 ^a^	0.41 ± 0.43 ^a^
*A. caliginosa*	0.45 ± 0.81 ^b^	0.46 ± 0.51 ^ab^	0.47 ± 0.41 ^ab^	0.50 ± 0.36 ^a^	0.51 ± 0.39 ^a^	0.52 ± 0.58 ^a^

Values presented are means of three replicates with standard error (Tukey’s Studentized Range HSD test *p* = 0.05). Different letters a, b, c … in the same row (6 values) indicate significant difference.

**Table 5:** phenanthrene Bioaccumulation factor (BAF), uptake rate (*k*_1_) and elimination rate (*k*_2_) constants in the both earthworms exposed to different phenanthrene concentrations.

Treatments	*Eisenia fetida*			*Aporrectodea caliginosa*		
	BAF	*k*_1_ (d^−1^)	*k*_2_ (d^−1^)	BAF	*k*_1_ (d^−1^)	*k*_2_ (d^−1^)
**P5**	0.42 ± 0.3 ^a^*	0.02 ± 0.06 ^c†^	0.05 ± 0.02 ^bc†^	0.47 ± 0.3 ^a^*	0.03 ± 0.06 ^ab†^	0.06 ± 0.02 ^d†^
**P10**	0.23 ± 0.2 ^ab^*^*^	0.016 ± 0.02 ^c‡^	0.07 ± 0.01 ^bc†^	0.31 ± 0.2 ^ab^*	0.031 ± 0.06 ^ab‡^	0.10 ± 0.03 ^c†^
**P15**	0.22 ± 0.3 ^ab^*	0.02 ± 0.09 ^c‡^	0.09 ± 0.03 ^b†^	0.27 ± 0.1 ^ab^*	0.03 ± 0.02 ^ab‡^	0.11 ± 0.02 ^c†^
**P20**	0.21 ± 0.3 ^ab^*	0.25 ± 0.06 ^b†^	0.12 ± 0.02 ^b†^	0.25 ± 0.3 ^ab^*	0.03 ± 0.09 ^ab‡^	0.12 ± 0.03 ^c†^
**P25**	0.22 ± 0.1 ^ab^*	0.05 ± 0.03 ^c‡^	0.25 ± 0.03 ^a†^	0.25 ± 0.4 ^ab^*	0.07 ± 0.08 ^a‡^	0.27 ± 0.02 ^b†^
**P30**	0.22 ± 0.2 ^ab^*	0.6 ± 0.01 ^a†^	0.27 ± 0.05 ^a‡^	0.24 ± 0.5 ^b^*	0.09 ± 0.02 ^a‡^	0.39 ± 0.04 ^a†^

Values presented are means of three replicates with standard error (Tukey’s Studentized Range HSD test *p* = 0.05). Different letters a, b, c…in the same column (5 values), number of asterisk * in the same row (2 values (BAF only)) and different symbols ^†^ and ^‡^ in the same row (4 values) indicate significant difference.
